# Activation by SLAM Family Receptors Contributes to NK Cell Mediated “Missing-Self” Recognition

**DOI:** 10.1371/journal.pone.0153236

**Published:** 2016-04-07

**Authors:** Elisenda Alari-Pahissa, Camille Grandclément, Beena Jeevan-Raj, Georges Leclercq, André Veillette, Werner Held

**Affiliations:** 1 Ludwig Center for Cancer Research, Department of Oncology, University of Lausanne, Epalinges, Switzerland; 2 Department of Clinical Chemistry, Microbiology and Immunology, University of Ghent, Ghent, Belgium; 3 Department of Medicine, McGill University, Montréal, Québec, Canada; Karolinska Institutet, SWEDEN

## Abstract

Natural Killer (NK) cells attack normal hematopoietic cells that do not express inhibitory MHC class I (MHC-I) molecules, but the ligands that activate NK cells remain incompletely defined. Here we show that the expression of the Signaling Lymphocyte Activation Molecule (SLAM) family members CD48 and Ly9 (CD229) by MHC-I-deficient tumor cells significantly contributes to NK cell activation. When NK cells develop in the presence of T cells or B cells that lack inhibitory MHC-I but express activating CD48 and Ly9 ligands, the NK cells’ ability to respond to MHC-I-deficient tumor cells is severely compromised. In this situation, NK cells express normal levels of the corresponding activation receptors 2B4 (CD244) and Ly9 but these receptors are non-functional. This provides a partial explanation for the tolerance of NK cells to MHC-I-deficient cells *in vivo*. Activating signaling via 2B4 is restored when MHC-I-deficient T cells are removed, indicating that interactions with MHC-I-deficient T cells dominantly, but not permanently, impair the function of the 2B4 NK cell activation receptor. These data identify an important role of SLAM family receptors for NK cell mediated “missing-self” reactivity and suggest that NK cell tolerance in MHC-I mosaic mice is in part explained by an acquired dysfunction of SLAM family receptors.

## Introduction

NK cells identify diseased target cells using a dual receptor system, which is based on arrays of activating and inhibitory cell surface receptors. Many inhibitory receptors, including Ly49 receptors in mice, killer cell immunoglobulin-like receptors (KIR) in humans and CD94/NKG2A in both species, are specific for MHC class I (MHC-I) molecules. These receptors counteract NK cell activation as long as cells express MHC-I molecules. Aberrant cells, such as tumor cells or virally infected that have lost MHC-I molecules are more susceptible to NK cell mediated attack or “missing-self” recognition. Indeed, the absence of MHC-I molecules is sufficient to render otherwise normal cells susceptible to attack although that seems to be restricted to cells of hematopoietic origin [1–3]. These findings provide evidence that normal hematopoietic cells activate NK cells. However the activating ligands, which confer missing-self recognition have remained poorly characterized.

The absence of knowledge regarding the relevant activation receptors has also hampered progress in understanding NK cell tolerance. NK cells do not attack normal hematopoietic cells that express MHC-I thanks to the action of inhibitory receptors specific for MHC-I molecules. However not all NK cells express inhibitory MHC-I receptors [4]. These NK cells respond poorly to stimulation via several activating receptors [4, 5], indicating that tolerance is based on impaired NK cell activation signaling. Similarly, NK cell activation signaling is compromised when NK cells develop in the complete absence of MHC-I. However, the activation receptors commonly tested do either not recognize normal cells (e.g. NKG2D) [6] or it is not known whether normal cells express ligands (e.g. NK1.1), indicating that these receptors are of questionable relevance to understand NK cell tolerance to normal self-cells. The expression and function of relevant activation receptors i.e. those mediating NK cell activation in response to normal cells is currently not known.

Key insights into the reactivity and tolerance of NK cells have been obtained using MHC-I deficient and transgenic mice [7, 8]. For example, NK cells from H-2^b^ mice do not reject syngeneic spleen cells while H-2D^d^ (D^d^) transgenic H-2^b^ mice (termed D^d^ mice) acquire the capacity to reject H-2^b^ cells [8]. When D^d^ is selectively deleted from T cells, NK cells in D^d^ mice fail to reject H-2^b^ targets [9, 10]. Such MHC-I mosaic mice provide a useful tool to investigate NK cells in hosts harboring cells with distinct haplotype. Clinically relevant situations include human leukemia patients that are reconstituted with (semi) allogeneic hematopoietic stem cells. In the above MHC-I mosaic mice, NK cells fail to reject T cells lacking D^d^, implying that activating receptors specific for ligands expressed by normal T cells are not functional. However, as indicated above, relevant activating ligands have remained poorly characterized.

We considered a role for Signaling Lymphocyte Activation Molecules (SLAM) family receptors as possible mediators of “missing-self” recognition since SLAM family members are solely expressed on hematopoietic cells. SLAM family molecules include SLAM (CD150, *Slamf1*), CD48 (*Slamf2*), Ly9 (CD229, *Slamf3*) 2B4 (CD244, *Slamf4*), CD84 (*Slamf5*), Ly108 (or NK-, T- and B-cell antigen (NTBA) in human) (CD352, *Slamf6*) and CRACC (CD2-like receptor activating cytotoxic cells) also termed CS1 (CD319, Slamf7). They generally mediate homotypic interactions, except 2B4, which recognizes CD48 (for review see [11]). Engagement of SLAM family receptors with ligands ectopically expressed on target cells has provided evidence that certain receptors activate wild type NK cells [12]. In addition, 2B4/CD48 interactions promote NK cell—NK cell contacts, which enhance NK cell function [13, 14]. Paradoxically, however, the analysis of NK cells from 2B4-deficient mice suggested that 2B4 is mainly an inhibitory rather than an activating receptor [15]. On the other hand the function of CRACC-deficient NK cells was reduced, indicating that CRACC is an activating NK cells receptor [16]. Redundancy and/or opposing roles of SLAM family receptors may mask their overall importance for target cell recognition. This issue has been difficult to address using classical gene knock out since Slam family genes are tightly linked on mouse chromosome 1. Indeed, T cells, B cells and NK cells express multiple SLAM family molecules [12]. Here we have addressed a possible redundant role of SLAM family molecules for the activation of wild type NK cells in response to “missing-self” targets. We further tested the functionality of relevant receptors in NK cells from MHC-I-deficient and mosaic mice to see whether their activity can explain self-tolerance.

## Materials and Methods

### Mice

C57BL6 (H-2^b^) and K^b^ D^b^ knock out mice were purchased form Harlan OLAC and Taconic, respectively. Floxed H-2D^d^ (on a C57BL6 background (D^d^) [9], CD4-Cre [17] and CD19-Cre [18] transgenic mice have been described before. D^d^ CD4-Cre and D^d^ CD19-Cre mice were obtained by breeding. Mice were housed under SPF conditions in individually ventilated cages. Animal experiments were conducted based on procedures approved by the Service Vétérinaire du Canton de Vaud (#1024.6) and performed in strict accordance with the recommendations in the Guide for the Care and Use of Laboratory Animals of the National Institutes of Health. Mice were euthanized by CO_2_ inhalation.

### Cell lines and CRISPR

B16 melanoma cells (H-2^b^), stably transfected with the indicated SLAM family members or an empty pSRɑ control plasmid [12] were provided by A.V. RMA cells (H-2^b^) originated from a Rauscher virus–induced C57BL/6 T-cell lymphoma [19] and RMA-S, a subline of RMA with low MHC class I surface expression [20], were electroporated with CRISPR vectors together with a plasmid encoding GFP at a ratio of 10:1. GFP+ cells were flow sorted after 24h and CD48 and Ly9 negative cells were obtained by Ab staining and flow sorting 6 days later.

For CRISPR the following sequences were cloned into Lenti CRISPR v2, a gift from Feng Zhang (Addgene plasmid # 52961) [21].

CD48: CACCGCCCTTGGGAACTGGATTTCAGTTT,

Ly9: CACCGATTCTTGGATTTTCAGAGAGGTTT

EGFP: CACCGGTGAACCGCATCGAGCTGAGTTT

### NK cell assays

Mice were primed by i.p. injection of 100 μg of polyinosinic-polycytidylic acid (poly I:C) (Invivogen) and spleens were harvested 24h later. Single cell suspensions were exposed for 4h to RMA or RMA/S cells, to confluent layers of B16 transfectants, or stimulated for 5h with NK1.1 mAb (PK136) coated plates or with phorbol 12-myristate 13-acetate (PMA) (50 ng/mL) and ionomycin (1 μg/mL). Lamp-1 (CD107) mAb was added at the initiation of the cultures and Golgi-Plug and Golgi-Stop was added 1 h later.

For rejection experiments mice were primed (as described above), 24h before injecting i.p a 1:1 mixture of control RMA (CFSE^hi^: labeled with 3.0 μM of CFSE (Molecular Probes) and the indicated type of RMA/S cells (CFSE^low^: labeled with 0.3 μM CFSE) (10^6^ cells total). Peritoneal cells were analyzed 20–24h later for the presence of transferred cells and the specific rejection was calculated as: 100 – [(%RMA output/ %RMA/S output) / (%RMA input / I% RMA/S input)] * 100).

Spleen cells from naive mice were passed through nylon wool columns to obtain combined NK cell plus T cell preparations. NK cells were purified using an NK cell enrichment kit (STEMCell). These cell preparations were cultured in complete DMEM supplemented with Glutamine, 10% FCS, 10 mM HEPES, 50 μM β-mercaptoethanol, and 0.5 μg/mL rhuIL-2 (a gift of N. Rufer, University of Lausanne). After 5 days, the purity of NK cells was around 50% for NK + T cell cultures and >80% for purified NK cells with <10% contaminating T cells. The cells were stimulated by addition to B16 transfectants as described above.

### Flow cytometry

Freshly isolated splenocytes or cultured cells were incubated with mAb 2.4G2 (CD16/32) hybridoma supernatant before staining with mixtures of biotin-labeled Ly49A (JR9-318) and a pool of FITC-labeled Ly49C/E (4D12: Note Ly49E is expressed by <1% of adult NK cells [22]; Ly49I (YLI90) and NKG2A/C/E (20D5) NK1-1-PercpCy5.5, CD3-APC and CD19-APCCy7, followed by streptavidine-AF700. SLAM expression was detected using PE-labeled antibodies to CD244 (0224F4), CD48 (HM48-1), Ly108 (13G3-19D), Ly9 (002) and CRACC (003), CD84 (mCD84.7) or Alexa-647 anti-SLAM (TC15-12F12-2) (Biolegend). MHC-I expression was detected using APC conjugated mAbs to H-2K^b^ (B8.24) and H-2D^b^ (B22/249). For intracellular staining, surface-labeled cells were fixed and permeabilized (Intracellular Fixation and Permeabilization Buffer Set) followed by staining with IFNγ (XMG1.2) mAb (eBioscience). Cells were run on a LSRII flow cytometer and analyzed with Flowjo10 software.

### Statistical analysis

For comparisons between two groups, statistical significance was determined using two-tailed Student *t* test with equal sample variance while a one-way ANOVA test with Bonferroni’s multiple comparison test was used for multiple comparison groups, as indicated in the figure legends.

## Results

### Expression of SLAM family receptors by lymphocytes

To address which SLAM family receptors contribute to the activation of NK cells by lymphocytes we determined their expression by NK cells as well as T and B cells from naive and poly(I:C) primed mice. NK cells from naive mice expressed high levels of 2B4, Ly9 and CD84, while Ly108 was expressed by a subset of NK cells and CRACC and SLAM were not detected **(S1 Fig,** data not shown and [12]). Priming expanded the Ly108 subset and induced CRACC expression on NK cells **(S1 Fig).** T cells and B cells from naïve and primed mice expressed high levels of CD48, Ly9, CD84, SLAM and Ly108, while CRACC was expressed at low levels on B cells, but not on T cells **(S1 Fig,** data not shown and [12]). Thus, NK cells express several SLAM family receptors that can serve as receptors for SLAM family members expressed by normal lymphocytes.

Since SLAM family receptors can exert diverse functional properties we next confirmed the ability of individual SLAM family receptors to activate NK cells. Primed NK cells from wild type mice readily released Lamp-1, produced IFNγ and robustly co-produced Lamp-1 and IFNγ in response to B16 cells stably transfected with CD48, Ly9 or CRACC (**S2 Fig**) in agreement with [12]. In contrast, we failed to see significant activation by Ly108 (**S2 Fig**), and CD84 had previously been shown to not activate NK cells [12]. Thus, combined with the expression analyses, normal T cells have the potential to activate NK cells using CD48-2B4 and Ly9-Ly9 interactions while activation by B cells may further involve CRACC-CRACC interactions.

### SLAM family receptors contribute to NK cell missing self-recognition

**Fig 1:**
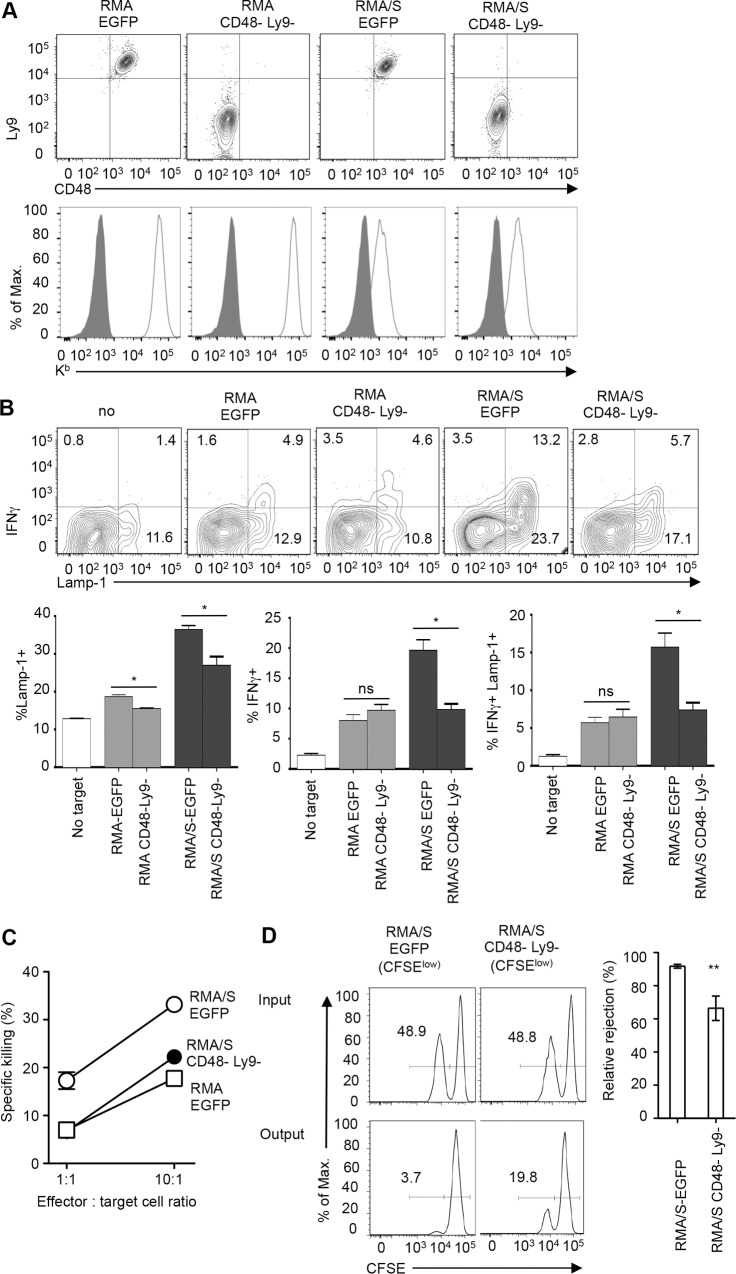
CD48 and Ly9 contribute to NK cell activation in response to a missing self target. **(A)** RMA and MHC class I^low^ RMA/S cells were transiently transfected with CRISPR vectors specific for CD48, Ly9 or EGFP and knock out cells were isolated by surface staining and cell sorting. Cells were stained for CD48, Ly9 and H-2K^b^. **(B)** Representative example of IFNγ production and Lamp-1 expression by primed NK cells in response to EGFP control and CD48 Ly9 double knock out RMA and RMA/S cells. The bar graph shows mean percentage (±SEM) of IFNγ+, Lamp-1+ and IFNγ+ Lamp-1+ NK cells following exposure to the indicated type of target cell. Statistics: unpaired t-test: * p< 0.05, ns not significant (p>0.05). **(C)** Killing (7-AAD staining) of EGFP control and CD48 Ly9 double knock RMA/S cells by primed NK cells *in vitro* (E:T), whereby spontaneous target cell death in the absence of effectors was subtracted. Data are means of triplicate determinations (±SD) from one experiment representative of 2 performed. **(D)** Mixtures of RMA cells (H-2^b high^) and RMA/S cells (either H-2^b low^ CD48+ Ly9+ or H-2^b low^ CD48- Ly9-, which had been labeled with a high and a low concentration of CFSE, respectively, were injected i.p. into primed H-2^b^ mice. Numbers in histograms depict the relative abundance of CFSE^low^ cells in the input mix and in the peritoneum of recipient mice 20 h later. The bar graph shows the mean percentage of rejection (±SEM) of the indicated RMA/S line relative to RMA cells. Data shown are complied from 2 independent experiments with 3–5 mice per group and experiment (total n = 8–9). Statistics: unpaired t-test, ** p< 0.01.

To address the importance of SLAM family receptors for missing-self recognition we used H-2^b low^ RMA/S thymoma cells, which activate NK cells. These cells serve as an appropriate model for missing-self recognition since parental RMA cells, which are H-2^b+^, are resistant to NK cells. Similar to normal T cells, both cell lines expressed high levels of CD48, Ly9 and CD84 while CRACC, Ly108 and SLAM were very low or absent **(S1 Fig**). To test whether CD48 (*Slamf2)* and Ly9 (*Slamf3)* contribute to NK cell activation, we disrupted the respective genes in RMA/S and parental RMA cells using CRISPR technology (**Fig 1A**). Loss of CD48 and Ly9 expression did not alter the expression of H-2K^b^ (**Fig 1A**) or H-2D^b^ or induce SLAM family members that are normally not expressed by these cells, such as CRACC (not shown). When NK cells from primed mice were exposed to RMA/S cells lacking CD48 and Ly9 the production of IFNγ and release of Lamp-1 was significantly reduced as compared to stimulation with RMA/S control cells (**Fig 1B**). NK cell mediated lysis of RMA/S cells lacking CD48 and Ly9 was also reduced (**Fig 1C**). Further, *in vivo* experiments showed that the rejection of RMA/S cells lacking CD48 and Ly9 was significantly lower than that of RMA/S control cells (**Fig 1D**). Inactivation of CD48 and Ly9 in parental RMA cells resulted in further reductions in the already low NK cell activation (**Fig 1A–1C**), indicating that CD48 and Ly9 also contribute to NK cell activation in the case of MHC-I-expressing cells. We conclude that CD48 and Ly9 significantly contribute to NK cell activation in response to a classical “missing-self” tumor target cell.

## The function of SLAM family receptors is influenced by MHC-I recognition

**Fig 2:**
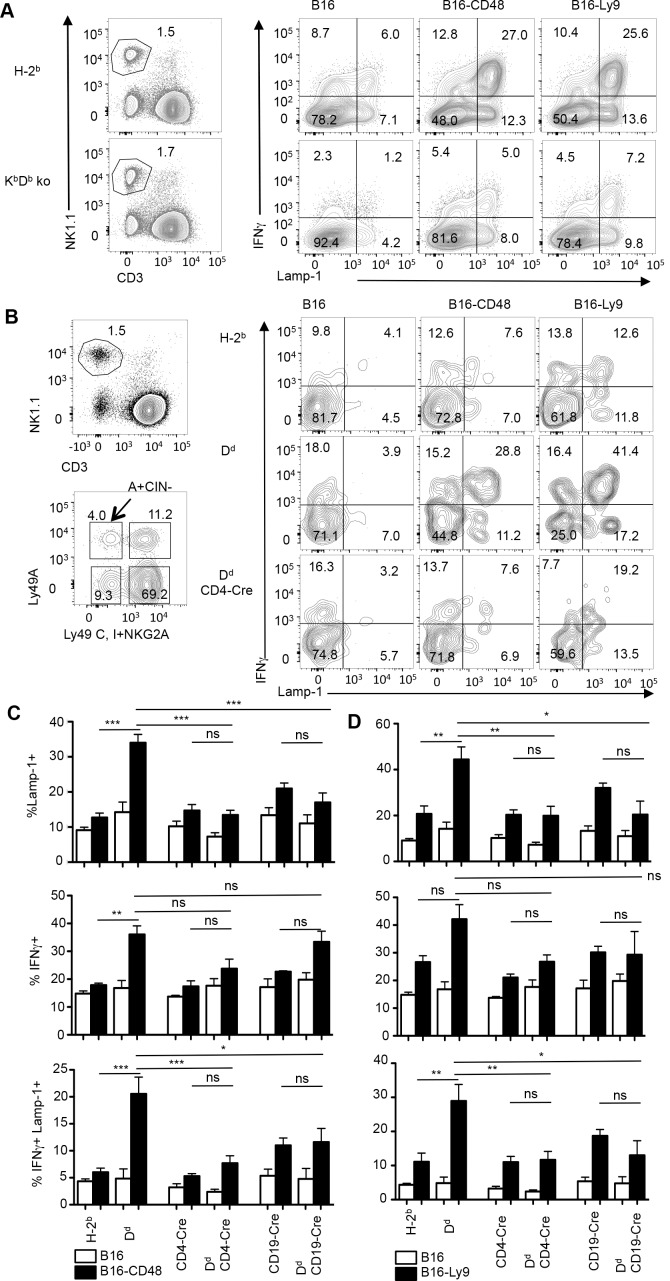
The activation function of CD48 and Ly9 depends on MHC class I recognition. **(A)** Splenocytes from primed B6 and K^b^ D^b^ knock out mice were added to B16 cells stably transfected with CD48 or Ly9 before analyzing the production of IFNγ and the release of Lamp-1 by NK cells. Data are representative of 2 determinations. **(B)** Splenocytes from primed H-2^b^ mice (top row), D^d^ mice (middle row) D^d^ CD4-Cre mice (T cell-specific D^d^ deletion) (bottom row) were exposed to B16 cells expressing a control plasmid (B16) or B16 cells stably transfected with CD48 or Ly9 cDNA. Splenocytes were harvested and NK cells expressing Ly49A and lacking Ly49C, Ly49I and NKG2A (A+CIN-) were analyzed for their production of IFNγ and expression of cell surface of Lamp-1. (**C**, **D**) The bar graphs show mean percentage (±SEM) of IFNγ+, Lamp-1+ or IFNγ+ Lamp-1+ among A+CIN- NK cells following exposure to B16 cells (open bars) or B16 cells expressing CD48 (**C**) or Ly9 cDNA (**D**) (black bars) in 3 independent experiments using 1–2 mice in each experiment (total n = 3–6). Statistics: One-way ANOVA *p<0.05, **p<0.01, ***p<0.005, ns not significant (p>0.05).

The functionality of activating receptors depends on the NK cell’s ability to sense MHC-I using inhibitory receptors [4, 5]. However, the activating receptors commonly tested in these assays, are either of unknown relevance (NK1.1) or are not relevant (NKG2D) for the recognition of normal lymphocytes [6]. We thus addressed whether the function of SLAM receptors, which are relevant for the recognition of normal lymphocytes, is influenced by MHC-I recognition. Indeed, as compared to NK cells from H-2^b^ mice, NK cells from K^b^D^b^-deficient mice responded poorly to stimulation by B16 cells expressing CD48 or Ly9 (**Fig 2A**), indicating that the function of SLAM family receptors was controlled by MHC-I expression. We further investigated the function of 2B4 and Ly9 on NK cells from MHC-I-expressing mice. NK cells expressing Ly49A (an inhibitory receptor for H-2D^d^) and lacking Ly49C, Ly49I and NKG2A (inhibitory receptors for MHC-I molecules expressed in H-2^b^ mice) (termed hereafter A+CIN- NK cells) recognize an MHC-I molecule in D^d^ but not in H-2^b^ mice. A+CIN- NK cells from H-2^b^ mice were inefficient at releasing Lamp-1 or producing IFNγ in response to B16 cells expressing CD48 or Ly9, while those from D^d^ mice responded efficiently (**Fig 2B–2D**). A-CIN+ NK cells, which recognize H-2^b^ molecules present in both mouse strains, responded equally efficiently to B16 CD48 cells (**S3 Fig**). Thus 2B4 and Ly9 receptors respond efficiently to stimulation when NK cells can recognize MHC-I. The impaired function of 2B4 and Ly9 explains at least in part the tolerance of NK cells to normal cells when NK cells fail to recognize MHC-I.

We extended these analyses to NK cells from mice with MHC-I-deletion on selected lymphocyte populations. Consistent with the data shown above and reported before [10], A+CIN- NK cells from H-2^b^ mice respond poorly to RMA (H-2^b+^) and RMA/S cells (H-2^b low^), while those from D^d^ transgenic mice (on a H-2^b^ background) respond efficiently. However, the response was impaired in D^d^ mosaic mice in which D^d^ was selectively deleted from B cells (using CD19-Cre mediated ablation of the floxed D^d^ transgene) (**S4 Fig**). Similar data were previously obtained when D^d^ was deleted from T cells using a CD4-Cre transgene [10]. Since T cells and B cells express CD48 and Ly9, we tested the functionality of 2B4 and Ly9 when NK cells persisted in the presence of T cells or B cells lacking D^d^. In either case, the engagement of 2B4 or Ly9 resulted in poor Lamp-1 release and Lamp-1 IFNγ co-production by A+CIN- NK cells, while IFNγ production was less affected **(Fig 2B–2D**). The effect was specific as A-CIN+ NK cells, which recognize H-2^b^ molecules that are present on all cells in all mouse strains, responded equally efficiently to B16 CD48 cells (**S3 Fig**). The impaired function of 2B4 and Ly9 explains at least in part why NK cells in MHC-I mosaic mice do not reject B or T cells lacking inhibitory MHC-I.

**Fig 3:**
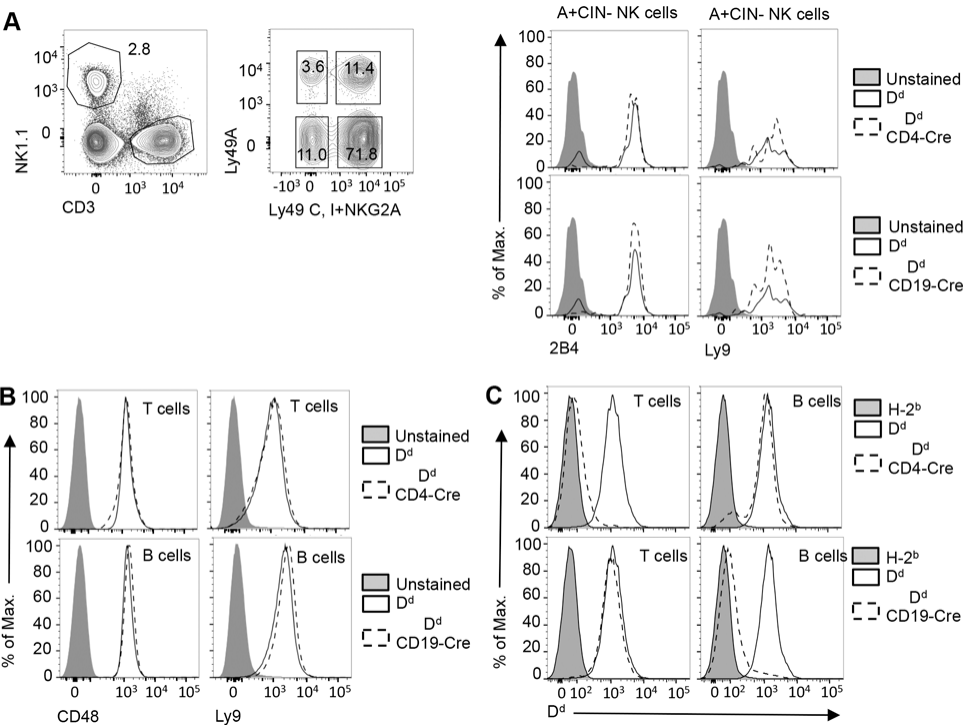
SLAM expression by NK cells is unaltered in D^d^ mosaic mice. **(A)** NK cells expressing Ly49A and lacking Ly49C, Ly49I and NKG2A (A+CIN-) from primed D^d^, D^d^ CD4-Cre (T cell specific D^d^ deletion) and D^d^ CD19-Cre mice (B cell specific D^d^ deletion) were analyzed for the expression of 2B4 and Ly9. **(B)** Histograms depict the expression of CD48 and Ly9 by T cells and B cells from primed D^d^, D^d^ CD4-Cre (T cell specific D^d^ deletion) and D^d^ CD19-Cre mice (B cell specific D^d^ deletion) mice. **(C)** Histograms show D^d^ expression by T cells and B cells from primed H-2^b^, D^d^, D^d^ CD4-Cre and D^d^ CD19-Cre mice, respectively.

We next addressed whether the inability to activate NK cells was associated with altered expression of SLAM family receptors. However, there was no difference in the expression of 2B4 or Ly9 on A+CIN- NK cells from primed H-2^b^, D^d^, D^d^ CD4-Cre and D^d^ CD19-Cre mice (**Fig 3A**). Moreover, as judged by a comparable CD69 up-regulation, there was no evidence of a difference in poly(I:C)-induced priming of A+CIN- NK cells, and these cells responded comparably to stimulation with PMA and Ionomycin (not shown and [10]), which indicates membrane proximal signaling defects. Finally, there was also no change in the expression of CD48 or Ly9 ligands on T or B cells from primed and naive D^d^ CD4-Cre and D^d^ CD19-Cre mice, respectively (**Fig 3B**). We conclude that the deletion of inhibitory D^d^ from T cells or from B cells does not impact the expression of SLAM family receptors on NK cells but strongly reduces the function of these receptors.

## The functional impairment of 2B4 is reversible

**Fig 4:**
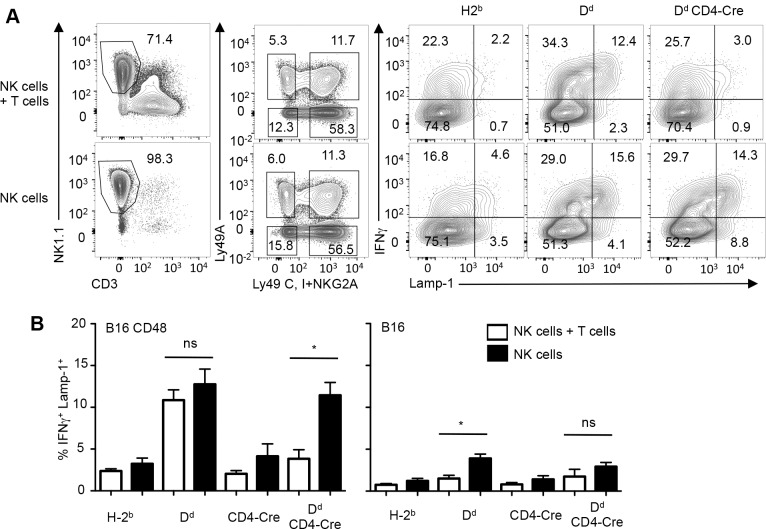
Restoration of 2B4 function on NK cells. (**A**) NK cells plus T cells (top row) or purified NK cells (bottom row) from naive B6, D^d^ and D^d^ CD4-Cre mice (T cell specific D^d^ deletion) were cultured in IL-2. After 5 days, cultures were exposed to B16 cells stably transfected with CD48. NK cells expressing Ly49A and lacking Ly49C, Ly49I and NKG2A (A+CIN-) were analyzed for the production of IFNγ and the expression of Lamp-1. (**B**) The bar graphs shows of A+CIN- NK cells from NK cell plus T cell cultures (open bars) or NK cell cultures (black bars) that are IFNγ+ Lamp-1+ following exposure to B16 cells stably transfected with CD48 (left) or to B16 cells expressing an empty control plasmid (right). Data depict means ± SEM from 2 independent experiments with 1 and 2 values per experiment (total n = 3). Statistics unpaired t-test *p<0.05, ns not significant (p>0.05).

We next tested whether the functional impairment of 2B4 was permanent or reversible. To address this issue we cultured NK cells from D^d^ CD4-Cre mice in the presence or absence of the autologous T cells and then determined 2B4 function on A+CIN- NK cells. When T cells remained present, A+CIN- NK cells from D^d^ mice responded efficiently to CD48 transfectants while those from H-2^b^ or from D^d^ CD4-Cre mice responded poorly (**Fig 4A and 4B**). When NK cells were cultured in the absence of T cells, A+CIN- NK cells from D^d^ mice responded efficiently while those from H-2^b^ mice still responded poorly to B16 CD48 cells. Thus, the responsiveness of cultured NK cells corresponded to that observed in the *ex vivo* analyses, independent of the presence of absence of T cells. In contrast, A+CIN- NK cells from D^d^ CD4-Cre mice that were cultured in the absence of T cells now responded as efficiently as A+CIN- NK cells from D^d^ mice (**Fig 4A and 4B**). Preliminary experiments indicate a corresponding restoration of Ly9 function when T cells are removed (not shown). Consistent with these data, when cultured in the absence of T cells, NK cells from D^d^ CD4-Cre mice recovered the ability to respond to RMA/S cells (**S5 Fig**). Thus, the removal of T cells, which lack inhibitory D^d^ and express activating CD48 and Ly9, was sufficient to restore the function of 2B4 and Ly9 receptors. NK cell tolerance in MHC-I mosaic mice is thus explained in part in part by an acquired dysfunction of SLAM family receptors.

## Discussion

Here we show that the murine 2B4 and Ly9 receptors significantly contribute to NK cell activation in response to classical “missing-self” tumor target cells. The activating function of these receptors is supported by the increased activation of NK cells using B16 cells transfected with CD48 or Ly9, in agreement with [12] and the reduced activation of NK cells in response to RMA/S cells that lack CD48 and Ly9 both *in vitro* and *in vivo*. Prior work has suggested that murine 2B4 in wild type NK cells is an inhibitory receptor [15]. This was based in part on the use of a spontaneous RMA/S variant that lacked CD48. It is possible that this variant differed in additional respects from parental RMA/S cells and that this contributed to the observed outcome. In addition, this latter study used prolonged (6–10 days) culture in IL2 to generate effector NK cells, which may modify the function of the 2B4 receptor. In contrast, we and others [12] used in vivo primed NK cell preparations and find an activating role of these receptors in wild type NK cells. Our data further reveal that there exist 2B4-Ly9-independent receptor/ligand interactions that contribute to the activation of NK cells by RMA/S cells. As these cells lack other SLAM family molecules known to activate NK cells it is possible that these latter activation signals are independent of SLAM family molecules. Based on our rejection experiments the latter mechanisms may be particularly important in vivo.

SLAM family receptors activate NK cells using small cytoplasmic SAP family adaptors (SLAM-associated protein) including SAP, EAT-2 and ERT. NK cells lacking SAP or all three SAP-family receptors are unable to mediate missing-self recognition [12]. In the absence of these adaptors, SLAM family receptors either fail to activate or undergo a switch-of-function and mediate inhibitory function. It was thus possible that these NK cells do no longer mediate missing-self recognition since SLAM family receptors inhibit the relevant NK cell activation receptors. As we removed activating SLAM family ligands from target cells we circumvent this caveat and show that defined SLAM family receptors contribute to NK cell activation in response to missing-self targets.

We further show that the responsiveness of 2B4 and Ly9 receptors is impaired when NK cells developing in the partial or complete absence of inhibitory MHC-I molecules in vivo. The impaired responsiveness of these receptors thus explains at least in part the tolerance of NK cells towards normal-self cells expressing the respective activating ligands CD48 and Ly9 while lacking inhibitory MHC-I. This hypo-responsiveness is not related to an altered expression of 2B4 and Ly9 but is likely based on membrane-proximal signaling defects since these NK cells respond normally to stimulation with PMA/Ionomycin. In addition, membrane-proximal signaling defects in hypo responsive NK cells have been shown in the case of the NK1.1 receptor [23], although the relevance of this receptor for the recognition of normal target cells is not known.

Three models can account for the MHC-I dependent changes of the function of activation receptor in NK cells [4, 5, 10, 24, 25]: In the absence of MHC-I, the activating 2B4/Ly9 self receptors may be responsible for tolerance induction via disarming i.e. chronic stimulation of NK cells via 2B4/Ly9 due to the lack of MHC-I–dependent inhibition eventually blunts the responsiveness of 2B4/Ly9. Consistent with this notion, chronic stimulation of NK cells via distinct receptors specific for non-self or stress-induced ligands has been shown to result in hyporesponsiveness [26–30]. Alternatively, it is possible that the responsiveness of 2B4/Ly9 is indirectly controlled, e.g. is based on an MHC-I dependent instructive mechanism that renders 2B4/Ly9 responsive to stimulation. Finally, it is possible that MHC-I recognition during NK cell development instructs NK cells to render their 2B4/Ly9 responsive to stimulation (arming) and then prevents the chronic activation of NK cells, which would reduce the responsiveness of 2B4/Ly9 (disarming). While the available data do not discriminate between these possibilities, the identification of receptors that are relevant for NK cell activation in response to normal cells should facilitate the investigation of the molecular mechanism(s) underlying NK cell reactivity and tolerance. Such investigations are important to better understand the functional properties of host-derived and donor-derived NK cells in human leukemia patients reconstituted with (semi) allogeneic hematopoietic stem cells.

## Supporting Information

S1 Fig Expression of SLAM by NK cells, T cells and B cells and tumor cell lines. **(A)** NK1.1+ CD3- (NK) cells, CD3+ T cells and CD19+ B cells present in the spleen of naive (open histogram) and poly I:C primed B6 mice (broken line) were analyzed for the expression of the SLAMs 2B4, CD48, Ly9 and Ly108 and CRACC as compared to unstained control samples (grey fill). **(B)** Analysis of SLAM expression on the indicated RMA and RMA/S variant (open histogram) as compared to unstained control samples (grey fill).
(TIF)

S2 Fig The function of SLAMs expressed by NK cells. **(A)** Analysis of SLAM expression on B16 transfectants (open histogram) as compared to B16 cells stably transfected with an empty control plasmid (grey fill). **(B)** Splenocytes from primed B6 mice were added to B16 cells stably transfected with the indicated SLAM before analyzing the production of IFNγ and the expression of Lamp-1 by gated NK cells. Bar graphs depict the production of IFNγ, the expression of Lamp-1 and the co-production of IFNγ and Lamp-1 by gated NK cells. Data represent means (±SEM) of 4–9 determinations from 3–5 independent experiments. Statistics: unpaired student’s t-test as compared cells stimulated with B16 control cells: ns not significant p>0.05, *p<0.05, **p<0.01, ***p<0.0001.
(TIF)

S3 Fig Differential responsiveness of NK cell subsets to CD48 transfectants. Splenocytes from primed H-2^b^, D^d^, D^d^ CD4-Cre (T cell-specific D^d^ deletion) and D^d^ CD19-Cre mice (B cell-specific D^d^ deletion) were exposed to B16 cells stably transfected with CD48 cDNA or an empty control plasmid (B16). Splenocytes were harvested and NK cells defiend by the differential expression of Ly49A versus Ly49C, Ly49I and NKG2A (A versus CIN) were analyzed for their production of IFNγ and expression of cell surface of Lamp-1. The bar graphs show mean percentage (±SEM) of IFNγ+, Lamp-1+ among A+CIN+, A-CIN+, A-CIN- and A+CIN- NK cells following exposure to B16 (open bar) or B16 cells expressing CD48 (black bars) of 3 independent experiments with 1–2 mice in each experiment. Statistics: One-way Anova *p<0.05, **p<0.01, ***p<0.005, ns not significant (p>0.05). Data for A+CIN- NK cells are identical to those shown in Fig 2C and are included here for comparison. While A-CIN- NK cells from B6 mice respond poorly A-CIN- NK cells from D^d^ mice respond efficiently to B16 CD48 cells. This is most likely due to the presence of Ly49G2+ NK cells among A+CIN- NK cells. While A+CIN+ NK cells from B6 mice respond efficiently A+CIN+ NK cells from D^d^ mice respond even more efficiently to B16 CD48 cells. This is consistent with the tuning model i.e. that the responsiveness increases with increasing inhibitory signaling input.
(TIF)

S4 Fig Impaired NK cell function in mice with B cell-specific D^d^ deletion. **(A)** Mixtures of H-2^b^ and D^d^ splenocytes, which had been labeled with a low and a high concentration of CFSE, respectively, were injected i.v. into primed H-2^b^, D^d^, CD19-cre and D^d^ CD19-Cre mice (resulting in B cell specific D^d^ deletion). Numbers in histograms depict the relative abundance of CFSE^low^ (H-2^b^) cells in spleens of the indicated recipient mice 24 h later. (**B, C**) The bar graphs show the mean percentage of rejection (±SEM) of H-2^b^ splenocytes (**B**) or of K^b^ D^b^ knock out splenocytes (**C**) relative to D^d^ splenocytes by the indicated strain of mice. Data are compiled from 4 (**B**) and 3 (**C**) independent experiments with 10 and 5 mice per point. Statistical significance: *** p< 0.001, ** p< 0.01. **(D)** Splenocytes from the indicated strains of primed mice were exposed to RMA cells (H-2^b^) for 4 h and NK cells (NK1.1+CD3-) expressing Ly49A but lacking Ly49C, Ly49I and NKG2A receptors (Ly49A+CIN-) were analyzed for the surface expression of Lamp-1 and the production of IFNγ. (**E, F**) The bar graphs show the mean percentage of Lamp-1+ IFNγ+ (±SEM) among Ly49A+CIN- NK cells from the indicated strains of mice following stimulation with RMA tumor cells (H-2^b^) (**E**) or plastic coated anti-NK1.1 (**E**). Data are from 1 experiment with two mice (E) and 3 independent experiments with 3–6 mice per point (**F**). Statistical significance: One-way Anova *** p< 0.001, ** p< 0.01.
(TIF)

S5 Fig Removal of D^d^-deficient T cells restores NK cell reactivity to RMA/S cells. Cultures containing NK cells plus T cells (**A, B**) or purified NK cells (**C, D**) from H-2^b^, D^d^ and D^d^ CD4-Cre mice were cultured in IL-2. After 6 days, cultured cells were either not stimulated (No) or exposed to RMA/S cells. NK cells expressing Ly49A and lacking Ly49C, Ly49I and NKG2A (A+CIN-) were analyzed for the production of IFNγ. The bar graphs show the percentage of IFNγ+ cells among A+CIN- NK cells. Data represent means (±SD) of 3 determinations from 2 independent experiments. Statistics: ns not significant (p>0.05), **p<0.01, ***p<0.005.
(TIF)
