# Supplementary figures and images for: Activation by SLAM Family Receptors Contributes to NK Cell Mediated “Missing-Self” Recognition
Source: PLoS One. 2016 Apr 7;11(4):e0153236. doi: 10.1371/journal.pone.0153236 (PMC4824421; doi:10.1371/journal.pone.0153236)

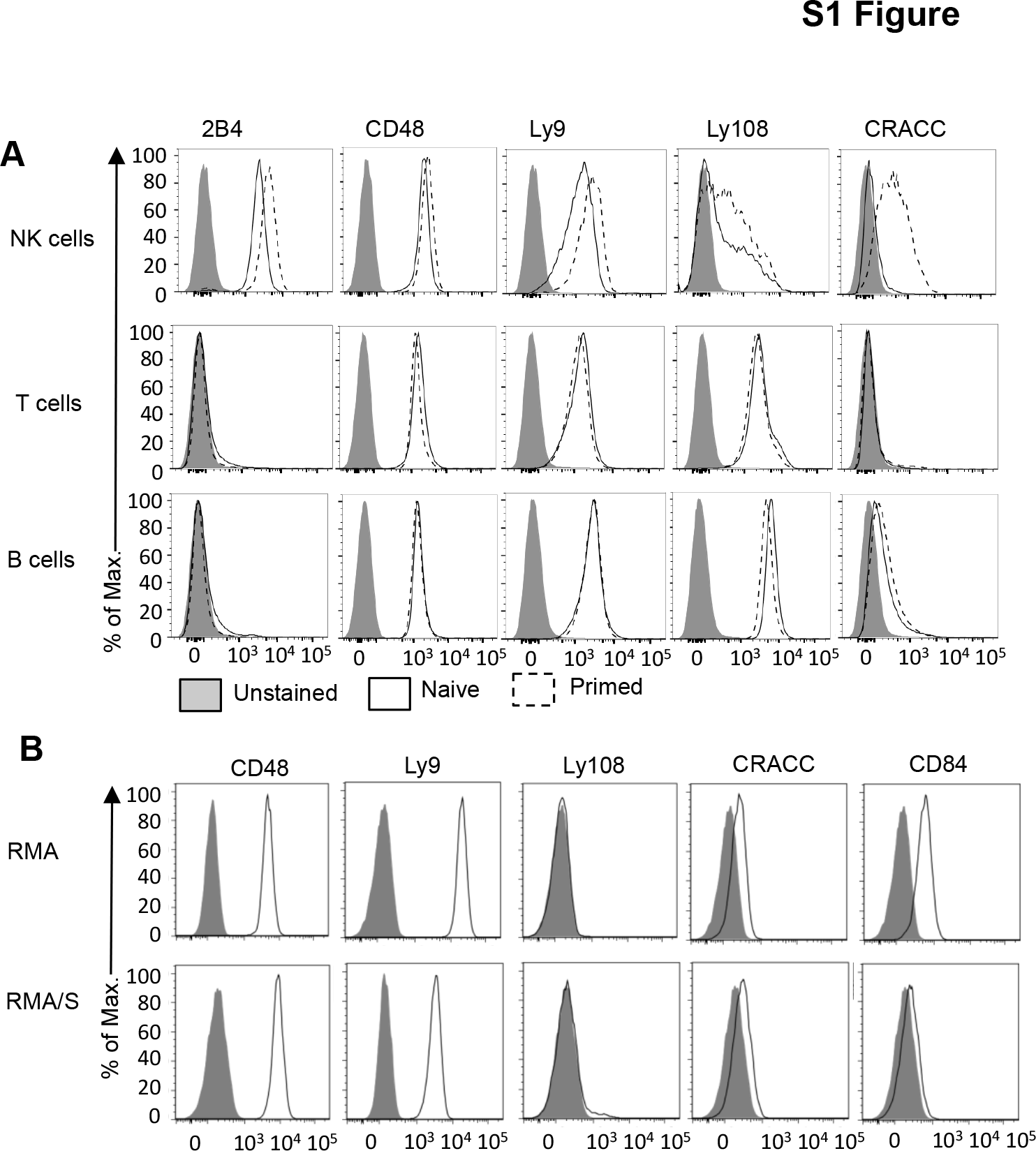

Supplement: S1 Fig — (A) NK1.1+ CD3- (NK) cells, CD3+ T cells and CD19+ B cells present in the spleen of naive (open histogram) and poly I:C primed B6 mice (broken line) were analyzed for the expression of the SLAMs 2B4, CD48, Ly9 and Ly108 and CRACC as compared to unstained control samples (grey fill). (B) Analysis of SLAM expression on the indicated RMA and RMA/S variant (open histogram) as compared to unstained control samples (grey fill). (TIF) [file pone.0153236.s001.tif]

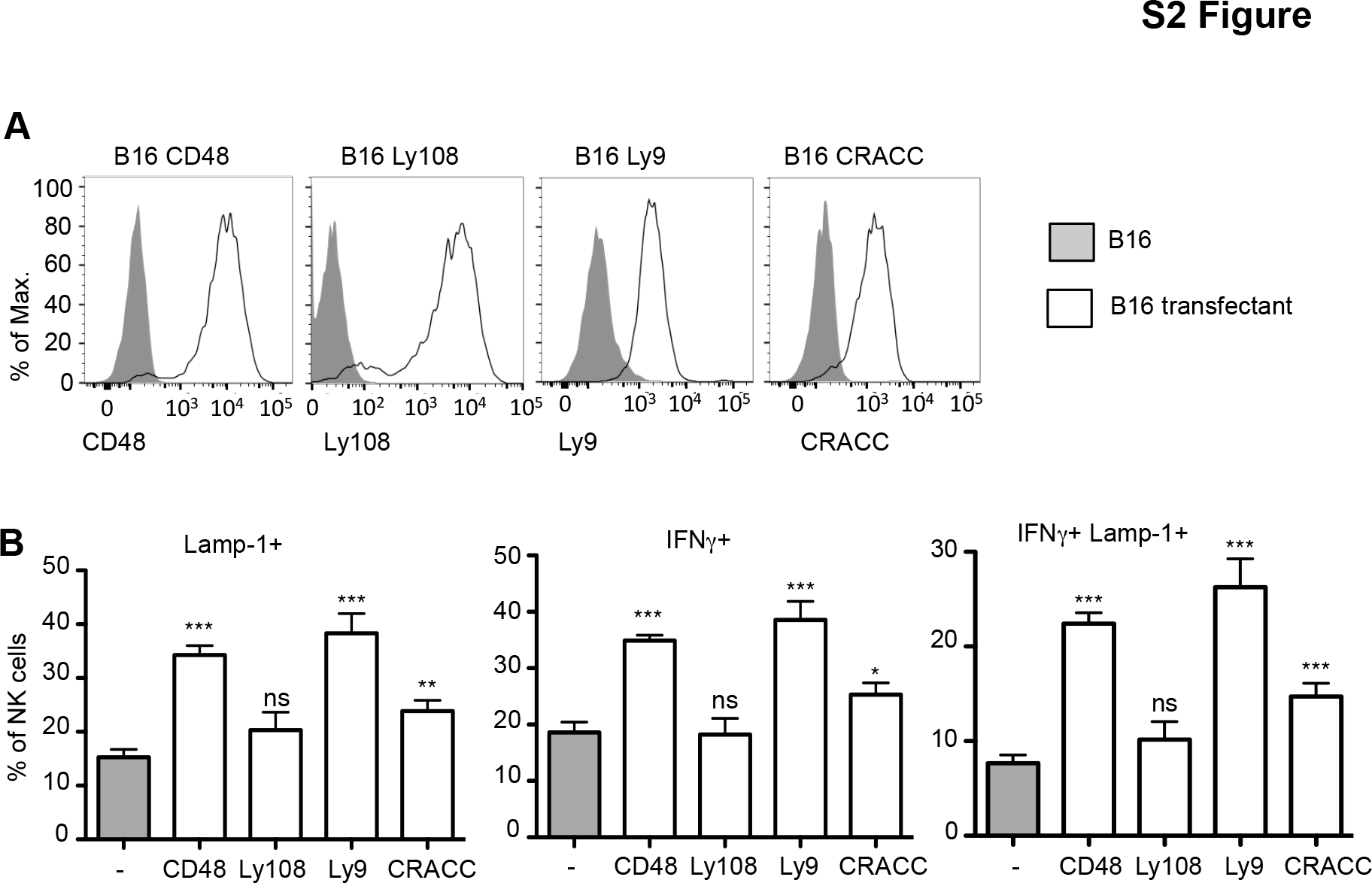

Supplement: S2 Fig — (A) Analysis of SLAM expression on B16 transfectants (open histogram) as compared to B16 cells stably transfected with an empty control plasmid (grey fill). (B) Splenocytes from primed B6 mice were added to B16 cells stably transfected with the indicated SLAM before analyzing the production of IFNγ and the expression of Lamp-1 by gated NK cells. Bar graphs depict the production of IFNγ, the expression of Lamp-1 and the co-production of IFNγ and Lamp-1 by gated NK cells. Data represent means (±SEM) of 4–9 determinations from 3–5 independent experiments. Statistics: unpaired student’s t-test as compared cells stimulated with B16 control cells: ns not significant p>0.05, *p<0.05, **p<0.01, ***p<0.0001. (TIF) [file pone.0153236.s002.tif]

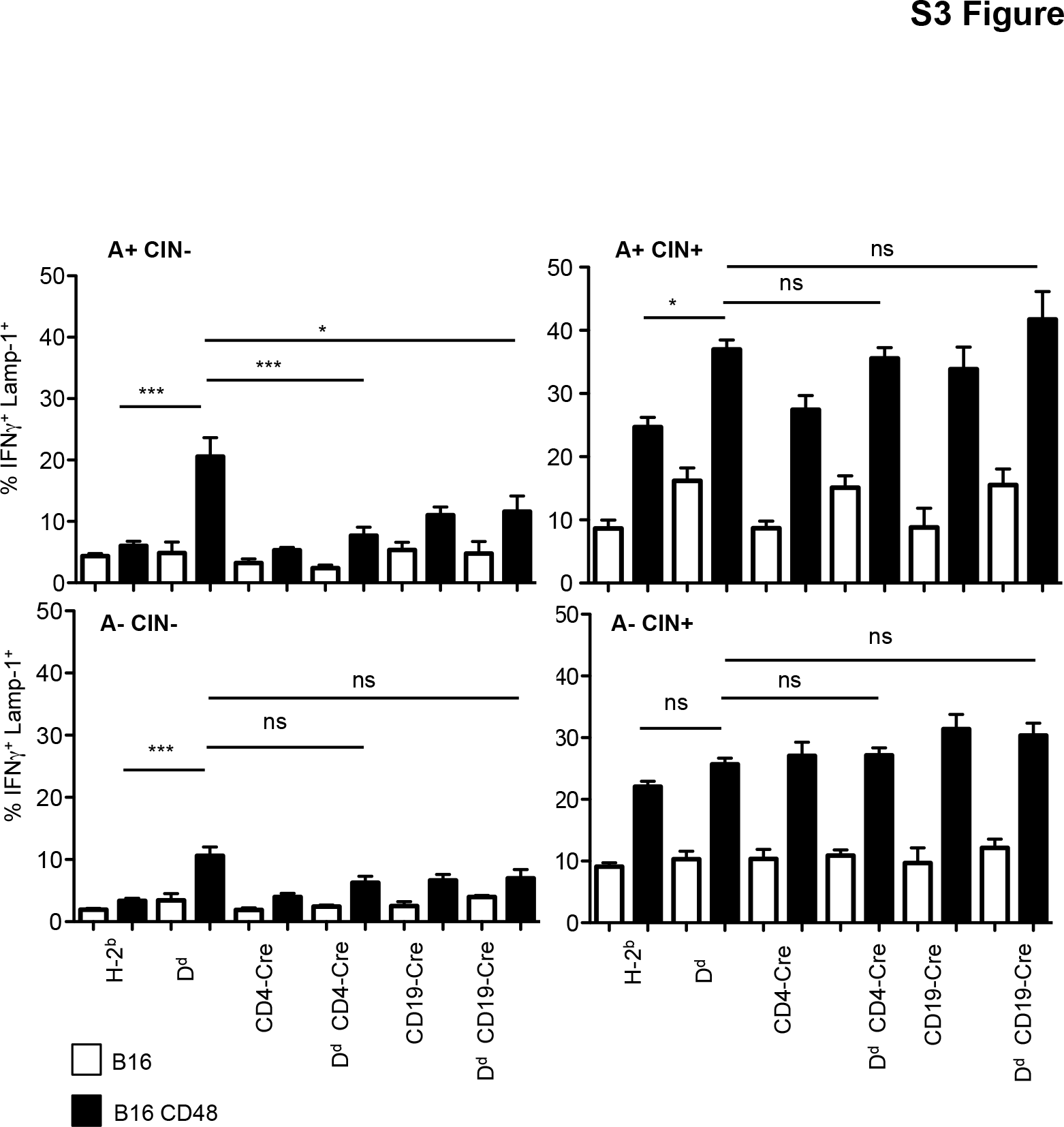

Supplement: S3 Fig — Splenocytes from primed H-2b, Dd, Dd CD4-Cre (T cell-specific Dd deletion) and Dd CD19-Cre mice (B cell-specific Dd deletion) were exposed to B16 cells stably transfected with CD48 cDNA or an empty control plasmid (B16). Splenocytes were harvested and NK cells defiend by the differential expression of Ly49A versus Ly49C, Ly49I and NKG2A (A versus CIN) were analyzed for their production of IFNγ and expression of cell surface of Lamp-1. The bar graphs show mean percentage (±SEM) of IFNγ+, Lamp-1+ among A+CIN+, A-CIN+, A-CIN- and A+CIN- NK cells following exposure to B16 (open bar) or B16 cells expressing CD48 (black bars) of 3 independent experiments with 1–2 mice in each experiment. Statistics: One-way Anova *p<0.05, **p<0.01, ***p<0.005, ns not significant (p>0.05). Data for A+CIN- NK cells are identical to those shown in Fig 2C and are included here for comparison. While A-CIN- NK cells from B6 mice respond poorly A-CIN- NK cells from Dd mice respond efficiently to B16 CD48 cells. This is most likely due to the presence of Ly49G2+ NK cells among A+CIN- NK cells. While A+CIN+ NK cells from B6 mice respond efficiently A+CIN+ NK cells from Dd mice respond even more efficiently to B16 CD48 cells. This is consistent with the tuning model i.e. that the responsiveness increases with increasing inhibitory signaling input. (TIF) [file pone.0153236.s003.tif]

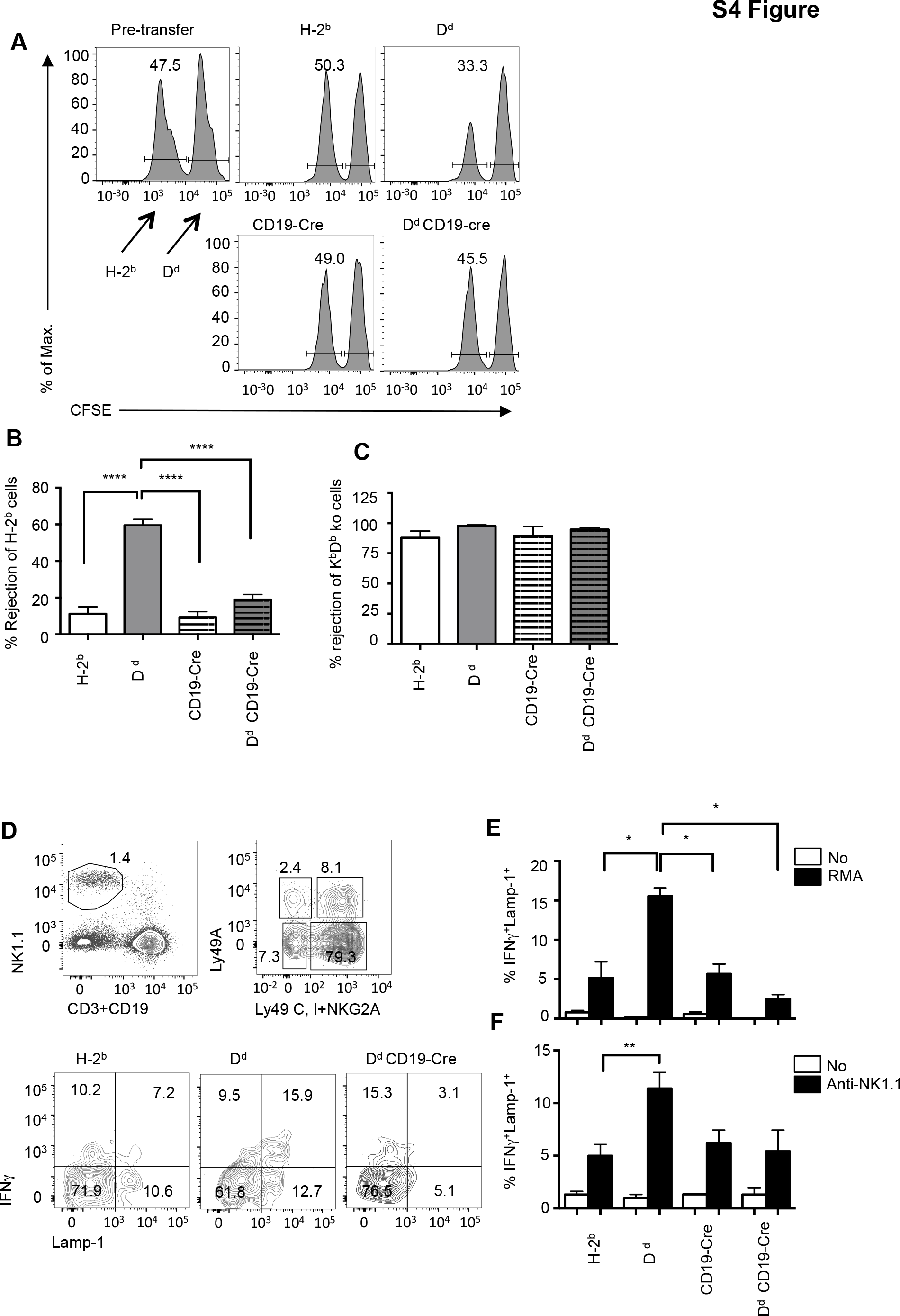

Supplement: S4 Fig — (A) Mixtures of H-2b and Dd splenocytes, which had been labeled with a low and a high concentration of CFSE, respectively, were injected i.v. into primed H-2b, Dd, CD19-cre and Dd CD19-Cre mice (resulting in B cell specific Dd deletion). Numbers in histograms depict the relative abundance of CFSElow (H-2b) cells in spleens of the indicated recipient mice 24 h later. (B, C) The bar graphs show the mean percentage of rejection (±SEM) of H-2b splenocytes (B) or of Kb Db knock out splenocytes (C) relative to Dd splenocytes by the indicated strain of mice. Data are compiled from 4 (B) and 3 (C) independent experiments with 10 and 5 mice per point. Statistical significance: *** p< 0.001, ** p< 0.01. (D) Splenocytes from the indicated strains of primed mice were exposed to RMA cells (H-2b) for 4 h and NK cells (NK1.1+CD3-) expressing Ly49A but lacking Ly49C, Ly49I and NKG2A receptors (Ly49A+CIN-) were analyzed for the surface expression of Lamp-1 and the production of IFNγ. (E, F) The bar graphs show the mean percentage of Lamp-1+ IFNγ+ (±SEM) among Ly49A+CIN- NK cells from the indicated strains of mice following stimulation with RMA tumor cells (H-2b) (E) or plastic coated anti-NK1.1 (E). Data are from 1 experiment with two mice (E) and 3 independent experiments with 3–6 mice per point (F). Statistical significance: One-way Anova *** p< 0.001, ** p< 0.01. (TIF) [file pone.0153236.s004.tif]

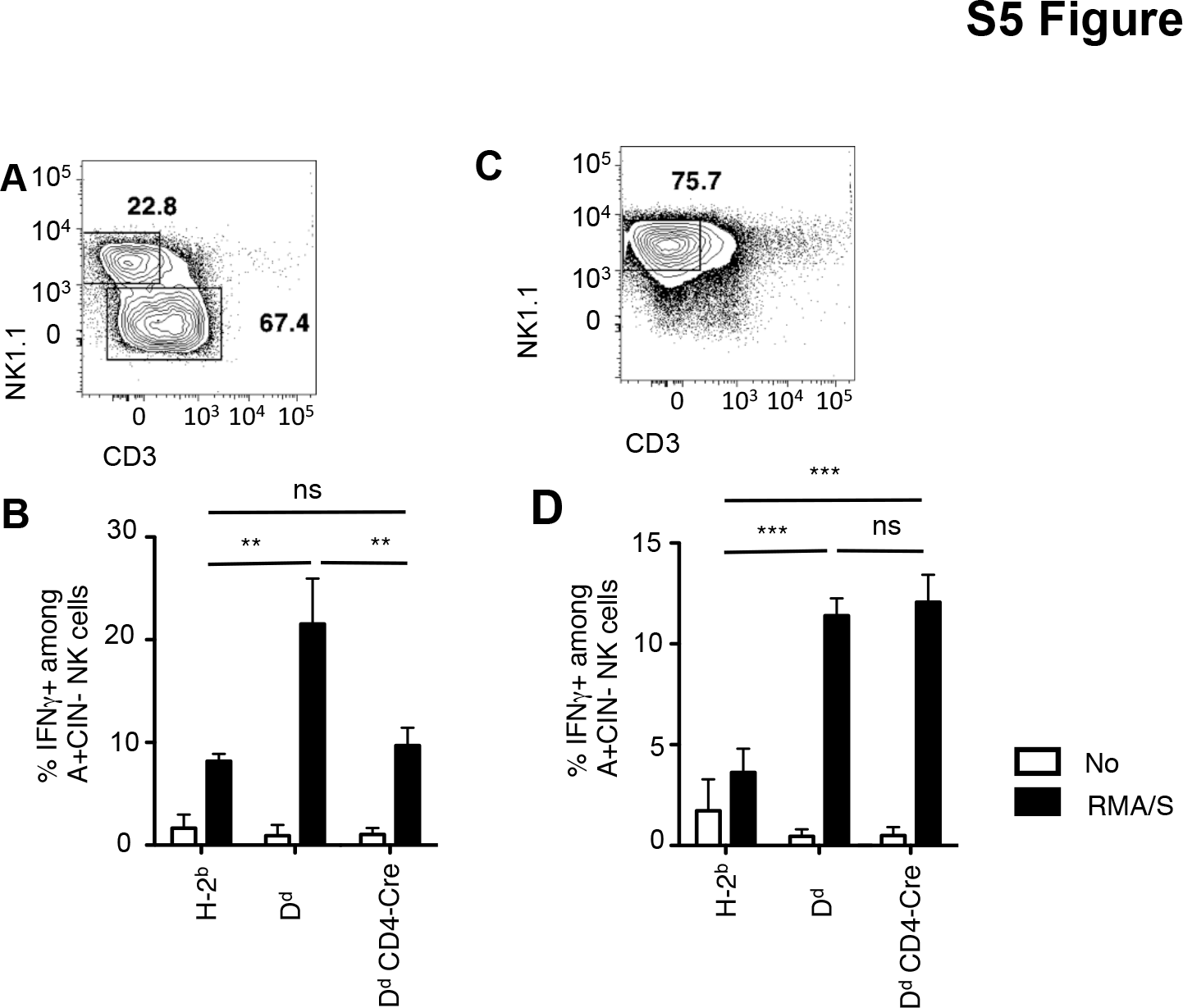

Supplement: S5 Fig — Cultures containing NK cells plus T cells (A, B) or purified NK cells (C, D) from H-2b, Dd and Dd CD4-Cre mice were cultured in IL-2. After 6 days, cultured cells were either not stimulated (No) or exposed to RMA/S cells. NK cells expressing Ly49A and lacking Ly49C, Ly49I and NKG2A (A+CIN-) were analyzed for the production of IFNγ. The bar graphs show the percentage of IFNγ+ cells among A+CIN- NK cells. Data represent means (±SD) of 3 determinations from 2 independent experiments. Statistics: ns not significant (p>0.05), **p<0.01, ***p<0.005. (TIF) [file pone.0153236.s005.tif]
